# Can ovarian aging be delayed by pharmacological strategies?

**DOI:** 10.18632/aging.101784

**Published:** 2019-01-23

**Authors:** Jinjin Zhang, Qian Chen, Dingfu Du, Tong Wu, Jingyi Wen, Meng Wu, Yan Zhang, Wei Yan, Su Zhou, Yan Li, Yan Jin, Aiyue Luo, Shixuan Wang

**Affiliations:** 1 Department of Obstetrics and Gynecology, Tongji Hospital, Tongji Medical College, Huazhong University of Science and Technology, Wuhan, Hubei, China

**Keywords:** organ senescence, ovarian aging, pacemaker, pharmacological strategies

## Abstract

Aging has been regarded as a treatable condition, and delaying aging could prevent some diseases. Ovarian aging, a special type of organ senescence, is the earliest-aging organ, as ovaries exhibit an accelerated rate of aging with characteristics of gradual declines in ovarian follicle quantity and quality since birth, compared to other organs. Ovarian aging is considered as the pacemaker of female body aging, which drives the aging of multiple organs of the body. Hence, anti-ovarian aging has become a research topic broadly interesting to both biomedical scientists and pharmaceutical industry. A marked progress has been made in exploration of possible anti-ovarian agents or approaches, such as calorie restriction mimetics, antioxidants, autophagy inducers etc., over the past years. This review is attempted to discuss recent advances in the area of anti-ovarian aging pharmacology and to offer new insights into our better understanding of molecular mechanisms underlying ovarian aging, which might be informative for future prevention and treatment of ovarian aging and its related diseases.

## Introduction

As human longevity has been significantly improved, aging-related problems are markedly increasing. It is predicted that the number of people over 60 years old by 2050 will be five times than that of 1950 [1]. As the world's most populous country, China entered into the aging society 13 years ago. According to the previous population census data, the aged population in China has exceeded world average in size, growth rate and proportion. The average lifespan of Chinese people will increase to 81.9 by 2040 [2]. The primary problem of the aging population is the serious detriments caused by the aging of various organs and the decline of their functions. Organ senescence is often highly associated with a variety of diseases, such as cancer, diabetes, cardiovascular disease and obesity. The occurrence and development of these diseases lead to the decrease of the life quality and increase of the proportion of people who live with the diseases. However, fortunately, aging has been shown to be an improvable condition, and delaying aging would be a way to prevent and treat diseases [3].

One of the earlier aging organs is ovary, as it exhibits an accelerated rate of aging compared with that of other body systems. Ovarian aging is characterized by gradual declines in ovarian follicle quantity and quality, ending with menopause [4]. The ovarian aging process is complicated and affected by a number of factors, including lifestyle, medical, genetic, autoimmune, environmental, and idiopathic ones. Thus, ovarian aging can be physiologic ovarian aging, which is defined by age-specific declines of functional ovarian reserve, and also premature ovarian failure (POF) due to those aforementioned factors. For women, anti-mullerian hormone (AMH )and antral follicle counts (AFC) are currently best markers for evaluating ovarian reserve. In addition, age and menstrual cycle are also good indicators. For animals, ovarian reserve is often reflected in follicle counts at all stages. Endocrine function is mainly reflected by hormone levels and estrous cycle regularity. Reproductive ability includes pregnant rate, litter size, number of offspring per litter, and so on.

Ovarian aging is a complex process. Since birth, a large number of follicles in the ovaries have undergone atresia during development. A woman only ovulates about 500 times in her lifetime, and 99% of the follicles are wasted. Rapid deterioration in both the ovarian follicle quality and quantity is highly associated with a number of women’ disorders or diseases. The fertility of women decreases gradually with age, and after age 35, it declines more rapidly until menopause at an average age of 51 [5]. Currently, more than 15% of couples in the world face the problem of infertility in their childbearing years, which is expected to reach 7 million by 2025 [6]. What’s more, estrogen secretion decreases with the decline of ovarian function and the arrival of menopause, which then lead to multiple organ dysfunction, such as heart disease, osteoporosis, cancer, obesity, senile dementia, and so on [7]. The incidence rate of osteoporotic fractures in postmenopausal women is significantly higher than that before menopause, and the risk index is much higher than that of men of the same age. In addition, cardiovascular diseases are often called "gender difference" diseases, because of their dramatic increase in postmenopausal women [8-10]. Thus, ovarian aging is considered as the pacemaker of female body aging, which drives the aging of multiple organs of the body [11].

Hence, it becomes particularly important to study molecular events underlying this fast aging process, as doing so would help us not only better understand this process, but also develop possible strategies or approaches to slow it down for hopefully preventing ovary-aging associated diseases. The past decade has witnessed a great progress in this area. This review is aimed to discuss recent advances in the pharmacological research toward development of anti-ovarian aging agents or approaches and to offer some insights into our better understanding of the ovary aging process and its molecular events. We hope that this review with a broad collection of literature would be informative and useful to ovary researchers and others who are interested in this topic. The subtitles and classifications in this article referred to one published article regarding anti-aging pharmacological strategies [12].

##  The free radical theory of aging and antioxidants

The free radical theory of aging has been a classical and the most influential theory in the field of aging, since was first postulated by Denham Harman in the middle of the last century. Oxidative stress leads to changes in the ovarian microenvironment, and these changes account for ovarian senescence and the decrease of ovarian reserve [13, 14]. During the senescence process, the level of reactive oxygen species (ROS) increases, while the expression and activity of oxidative defense system related enzymes are significantly reduced. These all lead to a wide range of oxidative damage, including the cell membrane lipid peroxidation, protein oxidation and enzymes inactivation and DNA damage [15-21]. Also, ROS in follicle oocytes, corpus luteum cells and follicular fluid of elderly women who receive assisted reproductive technology (ART) increases significantly, while antioxidant enzymes decreased; this causes the low success rate of ART [22-24]. It has been shown that oxidative damage products of DNA, protein and lipid in ovarian stromal cells increase markedly with age [25]. Clinical studies have also shown that higher ROS levels exist in unfertilized eggs or low-quality embryos [23, 26]. All these studies indicate that oxidative stress plays an important role in ovarian aging. Therefore, antioxidants have been used to prevent ovarian aging

### Vitamins C (ascorbic acid) and E (a-tocopherol)

Among all of the currently used antioxidants, vitamins C and E are commonly used as natural antioxidants. They are also the most studied ones. Vitamin C is the major water-soluble antioxidant, which can effectively reduce a-tocopheroxyl radicals and level of low-density lipoprotein (LDL) in cell membranes, thereby restoring a-tocopherol and inhibiting the generation of free radicals [27]. Vitamin E is the main hydrophobic antioxidant protecting cell membranes from oxidative damage by reaction with lipid radicals produced in the course of the lipid peroxidation chain reaction. The dietary vitamin E supplementation has been shown to be associated with reduced risk of atherosclerosis by reducing oxidative stress and inhibiting LDL oxidation [28]. These cellular studies are supported by an animal study, as oral administration of vitamins C and E could prevent the aging‐related negative effects on ovarian reserve (number of ovarian oocytes and number of ovulated oocytes) and oocyte quality (chromosomal aberration in metaphase II oocytes and morphological apoptotic oocytes) in mice [29]. Therefore, vitamins C and E can be useful for preventing ovary-aging.

### N-acetyl-L-cysteine

There are several lines of increasing evidence for benefits of using the antioxidant N-acetyl-L-cysteine (NAC) in preventing ROS-induced damage and pathology. Previous studies have shown that NAC effectively reduces oxidative stress-induced telomere shortening, telomere fusion and chromosomal instability in oocytes in vitro, and improves oocyte and early embryo development [30–32]. Another study using NAC in drinking water for mice for two months showed that NAC can improve the quality of mouse oocytes and promote the early embryonic development. By offering 1 to 1.5 months old mice with low doses of NAC for one year, this lab also found that NAC increases the litter size and oocyte quality of 7 to 10 months old mice and also telomerase activity and telomerase length [33]. These studies indicate that appropriate treatment with the antioxidants, such as vitamins C, vitamins E or NAC, can postpone ovarian aging by reducing free radicals.

### Curcumin

Curcumin is an active ingredient extracted from the dietary spice turmeric and has been utilized for medicinal purposes for thousands of years. The activity of curcumin as an antioxidant and free radical scavenger has been demonstrated by several studies [34]. Recently, more studies have demonstrated that curcumin could protect ovarian reserve and ovarian function from tissue injuries. Curcumin might be a promising treatment for cyclophosphamide-induced POF, because of its improvements in ovarian tissue histopathological damage, hormonal levels and reduced oxidative damages. It decreased vascular congestion and atretic follicles, increased healthy follicles numbers, and enhanced the levels of malondialdehyde (MDA), glutathione (GSH), superoxide dismutase (SOD), glutathione peroxidase (GPx), and catalase (CAT) in rat ovarian tissue [35]. In an ischemia-reperfusion rat model, curcumin could maintain and protect ovarian functions from oxidative injury [36]. Another study also indicated that curcumin can alleviate sodium arsenite-induced ovarian oxidative injury in a mouse model to a certain extent by increasing the levels of SOD and decreasing ROS and MDA expression [37]. Curcumin combined with flutamide could modulate ovarian structure and abdominal obesity in aging FSH-R haploinsufficient mice. In this model, curcumin enhanced the expression of bone morphogenetic protein-15 in the ovary and improved structural changes of in zona pellucida [38]. These studies indicate the beneficial outcomes of curcumin treatment in ovarian reserve and function protection.

### Coenzyme Q10

Coenzyme Q10 (CoQ10), an oil-soluble component of nearly all cell membranes, acts as an antioxidant in cellular metabolism via inhibition of lipid peroxidation, protein, and DNA oxidation [39,40]. CoQ10 is an essential component for transporting electrons in the mitochondrial respiratory chain to produce cellular energy. Studies have shown that supplementation of CoQ10 protects cells from ROS-induced damage due to its antioxidant properties, which strengthen endogenous cellular antioxidant systems. CoQ10 also restores oocyte mitochondrial function and fertility during reproductive aging. Thus, dietary administration of CoQ10 could prevent age-related decline in oocyte quality and quantity, and oocyte-specific Pdss2-deficient induced diminished ovarian reserve [41]. Another study indicate that CoQ10 supplementation may protect ovarian reserve by counteracting ovarian aging induced by either mal-functional mitochondria or physiological programming, and reducing the expression of 8’OHdG [42]. CoQ10 may restore ovarian function and reserve of aging mice, and more researches are needed to verify its effects.

### Proanthocyanidin

Proanthocyanidin is a phenolic compound exists in fruits, vegetables, nuts, seeds, wines and tea, which has been indicated to be effective in protecting tissues from oxidative damage [43]. Researchers have found that proanthocyanidin extracted from grape seed could maintain the homeostasis between cell proliferation and apoptosis, reduce oxidative damage in the D-gal-induced and natural aging ovaries, and alleviate D-gal-induced nucleus chromatin condensation, thus effectively delay the ovarian aging process in hens [44]. Currently, grape seeds have been widely produced as an antioxidant supplement and used for healthy living. More clinical trials are expected to demonstrate the role of proanthocyanidin in protecting ovarian function and delaying ovarian aging process in the human.

### Quercetin and other plant polyphenols

Some plant polyphenols can also protect ovarian reserve and ovarian function by acting as antioxidants. Quercetin is a potential antioxidant and free radical scavenger that is widely found in fruits, vegetables, and leaves. Quercetin increases the antioxidant capacity of the ovary in menopausal rats and in ovarian granulosa cell culture in vitro by upregulating the expression of some oxidative stress-related genes, such as SOD-1, CAT and glutathione synthetase (GSS) [45]. Some research suggested that tea polyphenols may inhibit the transition from primordial to developing follicles, extend the entire growth phase of a follicle and reduce dominant follicle numbers per cycle. Thus, tea polyphenols increased the reserve of germ cells, inhibited oocyte apoptosis and follicle atresia during ovarian development from birth to early aged, and retard climacterium in rats [46]. Drinking even a small cup of tea per day may extent the productive life of ovary.

Hence, these studies indicate that antioxidants can prevent ovarian aging and enhance ovary function by either activating the expression of genes important for reduction of oxidative damages or directly scavenging or inactivating ROS.

## Caloric restriction mimetics

Over the past years, it has been shown that improvement of cellular and physiological metabolisms could also prevent ovarian aging. One of the approaches is caloric restriction (CR), also known as dietary restriction (DR). CR by limiting the daily diet to 25% - 50% of the normal diet ensures that the body receives sufficient nutrients without malnutrition. The ability of CR to extend lifespan and delay aging have drawn noteworthy researchers’ attention since the original work was reported by McCay and colleagues eighty years ago [47]. Since then, a number of studies have confirmed that CR is the most effective means to postpone the aging of the body and extend lifespan of many organisms. Thereby it is the most inspiring discovery in the aging field [48]. CR can not only prolong the average life expectancy of rodents, but also improve the fertility and prolong the reproductive life [49], by delaying the process of ovarian aging. Although CR is so effective, its implementation is not so easy in real life practice. Thus, alternative substances have been looked for to mimic the CR effects as further detailed below. Also, the main signaling pathways that mediate the CR effects and the most promising pharmacological substances that modulate these pathways and mimic the CR effects will be discussed in the following sections.

### Regulators of glycolytic metabolism

Since CR can reduce the levels of insulin, blood glucose, and increase insulin sensitivity [50], inhibitors of the enzymes involved in the process of saccharide decomposition has been searched to simulate the CR-like effect on delaying aging [51]. One of the main inhibitors of this kind is metformin that has been used for the treatment of type 2 diabetes. Metformin has been shown to effectively improve the symptoms, such as insulin resistance, hyperinsulinemia, blood lipid metabolism. It can also prevent diabetes induced large blood vessels and pathological changes of capillaries, and then reverse early diabetes. Metformin may also act as a CR mimetic since it can decrease the production of hepatic glucose, by inhibiting of the mitochondrial respiratory- chain complex-1 [52]. Recently, metformin has become a hot research topic in the field of aging. In 2015, metformin was proposed for trials in delaying human aging [3]. The current studies of metformin in ovaries are mainly limited to polycystic ovary syndrome (PCOS) and ovarian cancer. Several studies have shown that metformin can improve ovarian function in patients with PCOS [52]. It could directly affect ovarian theca cells and decreased FSH-stimulated 3β-HSD, StAR, CYP11A1 and aromatase activities in both rat granulosa cells and women with PCOS, with reduction of basal and of FSH-stimulated progesterone and estradiol levels as a consequence [53]. However, results from another study showed that metformin has no significant effect on ovarian reserve [54]. Thus, the specific effect of metformin on ovarian reserve and function in normal animals or women needs a more systematic investigation.

### Inhibitors of insulin/IGF-1signal pathway

Previous studies have indicated that insulin-like growth factor-I (IGF-1) and its binding protein IGFBP-1 in follicular fluid may reflect ovarian reserve [55]. One small molecule inhibitor in metabolic enzymes, which might have a CR-like effect on ovarian aging is the synthetic glucose analog 2-deoxy-glucose (2-DG). Several studies have shown the activity and mechanisms of action of 2-DG for treating viral infection, epilepsy and cancer [56,57]. In mice, 2-DG treatment induced Foxo3 activity and inhibited primordial follicle activation, suggesting that 2-DG may be useful for fertility preservation [58].

Besides 2-DG, tamoxifen, an inhibitor of estrogen receptor (ER) often used for treatment of ER-positive breast and ovarian cancers [59,60] has also been shown to be potentially useful for improving ovarian aging. It was shown to regulate the IIS signaling involved in the occurrence of ovarian aging, and to keep the level of IGF-1 in the ovary [61]. An animal study also showed that tamoxifen can promote follicular development and AMH expression of and protect ovarian functions and the fertility of radiotherapy rats by enhancing the expression level of IGF-1 in ovaries [62].

### Inhibitors of mTOR pathway

Finally, regulation of the mammalian target of rapamycin protein (mTOR) has been shown to effectively simulate CR effects, extend lifespan, and delay aging [63]. As a serine/threonine protein kinase, mTOR regulates cell growth, proliferation, differentiation and the cell cycle [64]. Recent studies have shown that mTOR-related signaling pathways play an important role in aging associated metabolic diseases, such as obesity, type 2 diabetes and cancer [65]. Inhibiting the mTOR pathway can extend the life span of several species, such as worms [66,67], fruit flies [68] and mice [69]. Analysis of gene expression profiles indicated that the mTOR pathway is closely related to human health and life span [70]. Recently, rapamycin, an inhibitor of mTOR and a new type of macrolide immunosuppressant, has also been shown to inhibit the activation of the initial follicle by regulating the mTOR and sirtuin signaling pathways, thus protecting ovarian reserve and extending the reproductive life of the ovary [71–73]. Rapamycin may provide a new strategy for patients with premature ovarian failure by protecting ovarian reserve in the near future.

## Epigenetic regulators

Over the past few years, a series of epigenetic regulators have been identified, such as small molecule inhibitors of DNA methylation and histone acetyltransferases or non-coding RNAs. These regulators have the potency of treating cancer, myelodysplastic syndrome and neural degeneration [74]. Epigenetic regulations are involved in the changes of gene or protein expressions without altering DNA sequence. Epigenetic modifications are reversible. This characteristic makes small molecule epigenetic regulators attractive as aging intervention agents [75]. In the past 20 years, the role of histone and genomic epigenetic modification regulation in ovarian aging has been gradually recognized, and has become a research hotspot in this field. Abnormal regulation of the related gene expression can lead to ovarian cell apoptosis and accelerated aging, which in turn can accelerate the aging of the ovaries [76].

### Sirtuin activators and resveratrol

Although little is known about the role of small molecule regulators of DNA methylases and histone acetyltransferases in ovarian senescence, there are a few studies that suggest that some of them might be useful for improvement of ovarian aging. For example, the sirtuin deacetylase family with seven members in mammalian cells plays a very important role in cell survival, apoptosis, aging, and may be one of the common life-span control family genes in eukaryotic organisms. It was shown that a SIRT1 activator can increase ovarian reserve and prolong reproductive life of obese mice induced by high-fat diet by activating SIRT1 and inhibiting mTOR signaling pathway [77]. Also, resveratrol have been reported to protect mice from aging associated infertility by activating SIRT1 gene expression, improving the number and quality of oocytes, as shown by spindle morphology and chromosome alignment [78]. Resveratrol could improve the rat ovarian reserve and prolong the reproductive life, which may be due to inhibition of the activation of primordial follicle pool and follicular atresia [79]. These studies suggest that targeting epigenetic molecules might serve as an effective approach to improve ovary functions and to delay ovarian aging, though much more need to be done.

### MicroRNAs

MicroRNA profile changes in the ovaries of dwarf mice during the aging process, which suggests that these miRNAs may play vital roles in maintaining younger of ovarian phenotype [80]. Another study suggests that specific non-coding RNAs profiles are associated with age and ovarian reserve, indicating that oocyte quality might be mediated by ncRNA pathways [81]. However, there are no pharmacological strategies targeted at microRNAs to improve ovarian reserve or delaying ovarian aging process recently.

## Pharmacological induction of autophagy

Autophagy is an intracellular bulk degradation system. During the process, part of the cytoplasm is enveloped in the autophagosomes, then fused and degraded by lysosomes [82]. Several clinical trials are exploring autophagy as a therapeutic target as it plays vital roles in age-associated diseases [83].

In the ovaries, germ cell death is triggered by autophagy or apoptosis during the establishment of ovarian reserve [84]. Studies also suggest that autophagy may promote the formation of the primordial follicle pool [85], which also regulates ovarian follicle atresia [86]. However, few drugs delayed ovarian aging process by targeting autophagy signal pathway. Melatonin and Resveratrol are potential autophagy inducers that may extend ovarian lifespan. Melatonin is of quite capacity to delay ovarian aging for its regulation of multiple pathways such as oxidative stress, telomeres length and SIRTs, in which autophagy plays a vital role [87–89]. Furthermore, resveratrol improved the quality of oocytes by inducing autophagy and mitochondrial synthesis in aged cows [90]. The role of autophagy in ovarian aging process needs more research, and drugs target at autophagy should be explored later.

## Telomerase activators

Studies have indicated that telomere length are strongly associated with lifespan [91]. Reports on the role of the telomere and telomerase in female ovaries are still limited. Positive correlations were found between female reproductive life span, being widely accepted as ovarian reserve, and the telomere length [92]. The telomere length, serving as a biological clock, may play vital roles in limited ovarian lifespan, particularly at the cellular level [93]. Another study suggests that telomere length and telomerase activity are associated with primary ovarian insufficiency, which may indicate the progression of ovarian decline [94]. Compared to oocytes from young females, telomere length from the aged was remarkably shorter [95].

We have mentioned above that NAC could delay the oocyte aging by antioxidant activity. The telomerase activity and telomere length were also increased in the ovaries of mice treated with NAC [33]. Another study found that resveratrol protects against age-associated infertility in mice by enhancing telomerase activity and increasing telomere length in the ovaries [96]. These studies suggest that telomerase activators may extend ovarian lifespan effectively.

## Hormones

### Melatonin

Melatonin is a natural amine hormone secreted principally by pineal gland and released into circulation in a pulsatile fashion with the sharpest peaks in the early morning [97]. Light inhibits the secretion of melatonin and changes the time phase of melatonin rhythm. Different from the aforementioned antioxidants, melatonin possesses a variety of activities in the metabolic and physiological process. Melatonin exhibits direct free radical scavenging and indirect antioxidant effects. The main mechanism by which melatonin protects cell structure is to eliminate free radicals, inhibit the lipid peroxidation reaction and regulate activity of antioxidant and pro-oxidant enzymes. These actions of melatonin are believed to be mediated by the Keap1-Nrf2-ARE pathway and activation of sirtuin 1 (SIRT1).

The role of melatonin in preventing ovarian aging has been also reported. Previous studies have shown that shortening the time of sunshine can delay reproductive aging [98,99]. Researchers gave 10-day-old mice lifetime doses of melatonin and found that it delayed puberty arriving and the aging of the reproductive system, but had no effect on the size of the primordial follicle pool [100]. Another study showed that orally giving kunming mice at 2-3 months old age with melatonin for 12 months can significantly delay ovarian aging by increasing the total number of follicles and the number and quality of oocyte in mice, extending the telomere length, improving the fertility of old mice and reducing the ovaries the generation of ROS [101]. Melatonin treatment for 2 months can increase ovarian volume, improve estrous cycle, and maintain estrogen secretion of 13 month-old rat (middle aged) significantly, maintaining their hormone levels equivalent to that of young rats [102]. This study also showed that melatonin can extend the reproductive lifespan of the middle-aged and old rats. In a clinical study, perimenopausal and postmenopausal women of 42-62 years old were given melatonin treatment for six months. Amazingly, after the treatment, 43-49 years old women have significantly higher serum levels of LH, and their pituitary and thyroid function normally [103]. Finally, recent studies showed that melatonin can increase the primordial follicle pool size, delay ovarian aging in mice by enhancing antioxidant capacity, maintaining the telomerase activity, stimulating the SIRT1 expression and the ribosomal function [104]. These phenomena indicate that melatonin might promote the recovery of ovarian cycle and improve the elderly female fertility.

### Leptin

Hormones have been shown to play a role in ovarian ageing as well. For example, leptin has been demonstrated to be essential for ovarian follicle development and fertility. The expression of the leptin receptor in ovary is regulated throughout the estrous cycle by ovarian steroids, with peak expression at ovulation, indicating a possible involvement of this hormone in follicular development and corpus luteum formation. Also, IGF-I plays an important role in leptin receptor expression during the entire estrous cycle, especially during the prepubertal period [105]. During superovulation, leptin administration with gonadotropins in aged mice increased the ovarian response, developmental competence of oocytes and ovarian VEGF expression [106]. It was also shown that leptin causes an inhibitory effect on the early follicular development in both immature and adult mice, although the underlying inhibitory mechanisms of leptin may be different [107]. Another study indicated that reduction of peripheral leptin in the circulation promotes ovarian follicle development in prepubertal female mice, suggesting that leptin acts as an inhibitor of ovarian follicle development [108]. Leptin deficiency in mice was associated with impaired folliculogenesis, which resulted in increased follicular atresia. Thereby, leptin deficiency-induced follicle impairment may be one of the causative factor of infertility [109]. The recent study showed that neonatal overfeeding induces early decline of the ovarian reserve, and acute effects of elevated circulating leptin may be responsible for the long-term reproductive outcomes, thus leading to premature ovarian ageing and changes in reproductive efficiency [110].

### Dehydroepiandrosterone

Dehydroepiandrosterone (DHEA) is a hormone essential for human health, especially for women. All estrogens and nearly half of androgens are synthesized from DHEA in peripheral tissues. More studies have shown that DHEA can increase pregnancy rate, promote the steroid hormone secretion, increase the AMH expression and the number of antral follicles, which may also be due to the increased expression level of the IGF-1 [111]. Recent study indicates that DHEA supplementation prior to assisted reproductive technology (ART) improves ovarian markers such as serum AMH, inhibin B and antral follicle count (AFC) in patients with diminished ovarian reserve [112,113]. DHEA also has a pro-inflammatory immune potential and regulates the balance of CD4+/CD8+ T cells. Another study has also demonstrated that DHEA supplementation improves the ovarian reserve of women with diminished ovarian reserve which may due to its regulation of the immune response in the ovaries [114]. In a cortical autograft experimental sheep model, DHEA was applied for 10 weeks. The increased the expression of both the proliferation marker Ki-67 in granulosa cells and the follicular AMH expression at the preantral and early antral follicle stages was observed [115]. The results mentioned above suggest that DHEA may delay ovarian aging. However, long-term treatment with DHEA may affect folliculogenesis and lead to follicular atresia through interaction with AMH [116]. Thus, the concentration and duration of DHEA treatment is of great importance.

### Growth hormone

Growth hormone (GH) is a peptide which can promote cell proliferation and body development in animals and humans. GH has been applied to children and adults who suffers GH deficiency clinically. Mice lack of GH receptor showed the reduction of primordial follicle pool and the decrease of their survival rate of growing follicles. Results suggest that GH may be involved in the process of primordial follicle pool recruitment via the IGF-1 pathway, and inhibition of apoptosis of follicles [117]. GH can protect ovarian function from radiation therapy damage, promote the follicular development,and improve AMH expression by increasing IGF-1expression and repressing radiation-induced oxidative damage in ovary [118].

### Hormone replacement therapy

Hormone replacement therapy (HRT) is a universal treatment for premature ovarian failure (POF), mainly including estrogen therapy, estrogen and progesterone sequential therapy. Studies have shown that young POF patients treated with hormones show the development of secondary sex characters and alleviated perimenopausal syndrome [119]. Estrogen therapy can promote the endometrial proliferation in POF patients, maintain the estradiol concentration in the blood circulation and promote the pregnancy of women [120]. Estrogen and progesterone sequential therapy could enable POF patients to establish regular artificial cycles and improve pregnancy rate [121]. By comparing standard HRT treatment with percutaneous estrogen delivery combined with vaginal progesterone treatment, researchers found out that these treatments have similar effects on inhibition of FSH and LH, although the combination is more advantageous to cardiovascular protection [122]. Hence hormones-based treatments can be practically useful for improvement of ovary functions and delay of ovarian aging.

A recent study suggested that progesterone alone may protect ovarian function from ischemia-reperfusion injury through its anti-apoptotic and antioxidative properties [123]. More researches are still needed for the role of progesterone in ovarian function.

## Immunomodulators

A variety of autoimmune antibodies, such as anti-nuclear antibodies and anti-ovarian tissue antibodies, have been found to be related to POF [124]. Therefore, immunotherapy is of certain significance to patients with POF. In theory, immunomodulators are effective against autoimmune POF. It has been reported that patients treated with clinical immunosuppressive agents, such as glucocorticoid can resume ovulation and pregnancy, but the effect is not so clear. Administration of the immunosuppressant diethyleneethanol and freund's adjuvant during prepubertal provided sufficient anti-GnRH for calfs to delay the onset of puberty for 112 days [125]. Treatment with corticosteroids and testosterone can significantly improve the general condition of POF mice models by reducing the levels of lymphocytes, immune globulins, and related antibodies [126]. However, the clinical application of glucocorticoid has many adverse reactions and the long-term curative effect need to be followed-up. It was shown that the effect of androgens on autoimmune POF is significant, and their side effects are relatively less significant, so the patient's compliance is better. Androgens may inhibit the immune system by regulating the hypothalamic-pituitary-gonad axis [127]. Since the optimal treatment time and duration of POF with glucocorticoid or androgen have not been determined, further studies are needed.

## Traditional Chinese medicine

Traditional Chinese medicine holds that symptoms of perimenopausal women are related to the imbalance in the body of Yin and Yang, disturbance of viscera. Deficiency of the kidney energy is the fundamental cause, so it should be given priority to tonifying kidney and spleen, accompanied by protecting liver and calming nerves. Some can achieve satisfactory curative effect, such as Bushen Huoxue Recipe, Liu Wei Di Huang Jia Jian, Zuo Gui Wan Jia Jian and so on. The mechanisms of traditional Chinese medicine are complicated as the pharmacological ingredients are complex. Recent years, several studies have found that traditional Chinese medicine may regulate women nervous-endocrine system and immune system by removing free radicals, improving ovarian microcirculation and reducing cell apoptosis, and then delay ovarian aging [128,129]. The experiments of animals have revealed that, with age, the levels of nerve growth factor and norepinephrine (NE) in the ovaries, and the increase of sympathetic nervous activity were related to reproductive ability of rats. Electric acupuncture can promote the recovery of ovulation and increase fertility of older rats by reducing the level of NGF in ovary or blocking the activity of NE [130].

Intragastric administration of American ginseng gavage can protect the rat ovaries from VCD induced POF, promote the prostaglandin synthesis and ovulation, reduce the serum levels of PGE2, FSH and LH, remain the E2 to the normal level [131]. Bushen Huoxue Recipe alleviates cyclophosphamide-induced diminished ovarian reserve in mouse model by elevating the proportions of CD4+ T cells, Th1, Th17, Treg subsets and increasing serum levels of IFN-γ, TNF-α, IL-17A, IL-6 and IL-10 as well as the mRNA expressions of T-bet. These results show that Bushen Huoxue Recipe is a promising candidate to treat diminished ovarian reserve mice and this beneficial effect may be mediated through the downregulation of augmented autoimmunity [132]. Another study indicated that Bushen Huoxue Recipe might improve the ovarian reserve and enhance the ovarian function of POF mice through neo-oogenesis [133]. Bushen Tiaochong recipe might exert its beneficial role in oocyte maturation and restore diminished ovarian reserve through regulating the brain-derived neurotrophic factor pathway [134].

Yifuning, a traditional Chinese medicine recipe, has been used for many years in China, for its effects on treating climacteric syndrome. Studies suggest that it can improve the general condition of aging rats and mice, promote hormone secretion, recover estrous cycle, increase the viscera index of ovary and uterus and improve perimenopausal syndrome symptoms. It can also improve the function of natural aging reproductive organs such as ovary, uterus and vaginal, which may be related to its activities of antioxidant and promoting proliferation [135]. The Bushen compound could delay the ovarian aging of rats and significantly improve their reproductive and endocrine functions by reducing oxidative damage, promoting the production of VEGF, IGF-1 growth factor, reducing the level of TNF-α in the ovarian tissue, and effectively improving ovaries microenvironment. It can also improve the ultrastructure of ovarian tissue and enhance the immune function of the body [136]. Diosgenin improves ovarian reserve in naturally aging mice by increasing the number of primary follicles and serum levels of AMH. The mRNA expression of NOBOX, GDF9 and BMP15 was also evaluated [137]. Our recent study have also indicated that Kuntai capsule may improve damaged ovarian function, which may be related to its antioxidant and anti-apoptosis effects [138]. As the components of traditional Chinese medicine are complex, they may have several pharmacological actions and work through a variety of pathways. Traditional Chinese medicine shows a great potential in delaying ovarian aging and extending ovarian lifespan.

## Conclusions

**Figure 1:**
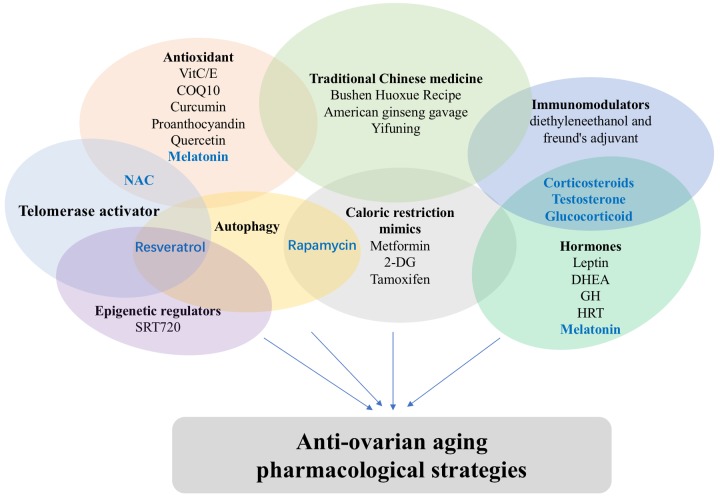
**Classification of anti-ovarian aging drugs.** Some drugs may have several pharmacological actions.

Nowadays, many young women choose to delay or avoid marriage and/or giving a birth owing to the accelerated pace of our society and various pressures from work and family. However, their ovarian reserves strictly define their best age of fertility, since a sharp decline in ovarian functions is observed after age 35, which defines a high-risk period. After 35 years-old, the rate of pregnancy markedly decreases while miscarriage and premature birth for a woman increase significantly. Consequently, the health of maternal females and child is threatened post this stage. Therefore, more systematic and careful studies of the female reproductive system, especially the mechanisms of ovarian aging and the effective delay strategies, are of great importance. Current research on ovarian aging mechanisms, such as primordial follicles activation and follicular atresia, have provided new insights into our better understanding of molecular mechanisms underlying ovarian aging delay. This rich information has been useful for development of anti-ovarian aging therapies as described above. Upon our review, some drugs may have several pharmacological actions and work through a variety of pathways (Figure 1). We believe that, upon future establishments of effective therapies, it will no longer be a fantasy to extend women's reproductive life and delay menopause with the development of medical advances in this specific area.
